# High-density genetic linkage mapping reveals low stability of QTLs across environments for economic traits in *Eucalyptus*

**DOI:** 10.3389/fpls.2022.1099705

**Published:** 2023-01-18

**Authors:** Xianliang Zhu, Qijie Weng, David Bush, Changpin Zhou, Haiwen Zhao, Ping Wang, Fagen Li

**Affiliations:** ^1^ Key Laboratory of National Forestry and Grassland Administration on Tropical Forestry Research, Research Institute of Tropical Forestry, Chinese Academy of Forestry, Guangzhou, China; ^2^ Commonwealth Scientific and Industrial Research Organisation (CRISO) Australian Tree Seed Centre, Canberra, ACT, Australia

**Keywords:** *Eucalyptus urophylla* × *tereticornis*, stable QTL, multi-environment trials, candidate genes, genotyping by sequencing

## Abstract

**Introduction:**

*Eucalyptus urophylla*, *E. tereticornis* and their hybrids are the most important commercial forest tree species in South China where they are grown for pulpwood and solid wood production. Construction of a fine-scale genetic linkage map and detecting quantitative trait loci (QTL) for economically important traits linked to these end-uses will facilitate identification of the main candidate genes and elucidate the regulatory mechanisms.

**Method:**

A high-density consensus map (a total of 2754 SNPs with 1359.18 cM) was constructed using genotyping by sequencing (GBS) on clonal progenies of *E. urophylla × tereticornis* hybrids. QTL mapping of growth and wood property traits were conducted in three common garden experiments, resulting in a total of 108 QTLs. A total of 1052 candidate genes were screened by the efficient combination of QTL mapping and transcriptome analysis.

**Results:**

Only ten QTLs were found to be stable across two environments, and only one (*qSG10Stable* mapped on chromosome 10, and associated with lignin syringyl-to-guaiacyl ratio) was stable across all three environments. Compared to other QTLs, qSG10Stable explained a very high level of phenotypic variation (18.4–23.6%), perhaps suggesting that QTLs with strong effects may be more stably inherited across multiple environments. Screened candidate genes were associated with some transcription factor families, such as TALE, which play an important role in the secondary growth of plant cell walls and the regulation of wood formation.

**Discussion:**

While QTLs such as *qSG10Stable*, found to be stable across three sites, appear to be comparatively uncommon, their identification is likely to be a key to practical QTL-based breeding. Further research involving clonally-replicated populations, deployed across multiple target planting sites, will be required to further elucidate QTL-by-environment interactions.

## 1 Introduction

Tree growth and wood properties are important traits in breeding programs for fast-growing forest trees including *Eucalyptus*. Quantitative trait locus (QTL) mapping has revealed important genetic regions that have influence on growth and wood property traits of major forest tree species (Hall et al., 2016; Mori et al., 2019). The interaction between growing environment and QTL expression and mapping in forest trees is complex, and gaining a better understanding of the phenomenon may have significant impacts on a forest tree breeding program (Rae et al., 2007). In crops planted in the field, mounting evidence suggests that the majority of QTLs do not regulate target traits across multiple environments (Shang et al., 2015; Raihan et al., 2016; He et al., 2021). Similarly, in forest trees, some studies have attempted to identify the stability of QTLs across multiple environments. For example, Westbrook et al. (2015) investigated QTLs regulating the stem resin channels by integrating genetic analyses across environments, ages, and populations of *Pinus taeda*. Mori et al. (2019) performed QTL mapping for growth and wood properties of *Cryptomeria japonica* across three environments. These studies both suggest that for long-lived forest species, QTLs for growth and wood property traits tend to be sensitive to environments.

Globally*, Eucalyptus* is one of the most important cultivated forest tree genera, providing fuelwood, fibre and timber (Ladiges et al., 2003). Due to its economic importance, QTL mapping for growth traits and wood properties has been intensively carried out (Gion et al., 2011; Bartholomé et al., 2013; Freeman et al., 2013). Some significant challenges to making practical use of QTLs have been identified (Grattapaglia and Kirst, 2008). Firstly, QTLs identified in study populations have not proven to be stable in eucalypt breeding populations. This is a particular issue for highly genetically diverse breeding populations that are managed by open pollination or large numbers of controlled-pollinated mating among members. The issue is less likely to be problematic where progeny of small sets of controlled pollinated crosses are made, for example to produce hybrid progeny from elite pure-species parents. This latter scenario is more likely in tropical regions, including major plantation growing regions such as Brazil and southern China, where hybrids and clonal selections are typically deployed (Grattapaglia and Kirst, 2008; Xie et al., 2017).

A second issue to making practical use of QTLs is that growth and wood property traits are quantitative (i.e., numerous genes of small effect are likely to contribute) and are subject to genotype-by-environment interactions (G × E) (Malan and Verryn, 1996; Brawner et al., 2013; Bush et al., 2015; de Araujo et al., 2019). Quantitative genetic studies have shown that G × E is more marked for growth traits than it is for wood properties (Downes et al., 1997), although both are significant. It is therefore not surprising that evidence for QTL × environment interactions has been found (Freeman et al., 2013; Westbrook et al., 2015; Mori et al., 2019), the problem being that QTLs identified from a study of a single site may not be identified at a second or subsequent sites. The challenge is therefore to find QTLs that are stable over multiple sites. Among these studies, Freeman et al. (2013) highlighted QTL stability in regulating growth and wood properties across different pedigrees and multi-environmental trials in *E. globulus* using control-pollinated families, however, the impact of environmental effects is likely to be harder to detect using non-clonal plants.

Despite the more-sophisticated analytical methods which are now available, classical genetic linkage maps remain important tools for QTL analysis and efficient programs for breeding (Li et al., 2020b). In recent decades, molecular markers such as SSR, DArT and SNP have been used to construct genetic maps for forest trees (Kullan et al., 2012; Pavy et al., 2017). Of these, SNP markers based on high-throughput techniques are gaining popularity due to the dramatically increased marker density that can be achieved per unit cost. Moreover, the cost of SNP genotyping continues to fall, with the development of high-throughput technology such as genotyping by sequencing (GBS) (Elshire et al., 2011). Recently, GBS technology has been widely applied to the genotyping of perennial woody plants, such as *Eucalyptus* (Brenton et al., 2019; Klápště et al., 2021), *Populus* (Carletti et al., 2016; Xia et al., 2018), *Cinnamomum* (Gong et al., 2021) and *Pinus* (Hirao et al., 2022), accelerating the molecular breeding progress in these species. The publication of the *E. grandis* reference genome (Myburg et al., 2014) has also significantly aided QTL mapping efforts, with high synteny between *E. grandis* and other common commercial eucalypts. In *Eucalyptus*, most of the previous QTL studies found few substantive associations between target traits and candidate genes due to the low resolution of genetic map, large QTL interval, or lack of reference genomic information (Freeman et al., 2009; Thumma et al., 2010; Freeman et al., 2013; Sumathi et al., 2018).

Due to advances in high-throughput sequencing technologies, researchers have conducted some transcriptome sequencing (RNA-seq) to discover functional genes and try to reveal molecular mechanisms of complex traits in *Eucalyptus* (Nakahama et al., 2018; Vaillant et al., 2018; Zhang et al., 2022). For example, Zhang et al. (2022) performed RNA-seq to identify the co-expressed genes during the callus maturation and shoot regeneration of *Eucalyptus*, and found a number of potentially functional genes associated with reproductive capacity. Studies in other woody plants, such as in *Picea glauca* (Laoué et al., 2021) and *Eriobotrya japonica* (Peng et al., 2022), have shown that the combination of QTL mapping and transcriptome data will significantly improve the efficiency of mining for functional genes and elucidating the genetic mechanism.

Although considerable progress has been made in dissecting the genetic mechanism of growth and wood property traits, much remains unknown in *Eucalyptus*. Economic trait QTLs, their stability and transcriptome activity across environments is an important area for future research. In this study, large-scale population GBS sequencing and small-scale RNA-seq on clonal progenies of *E. urophylla* × *tereticornis* hybrids were performed. We aimed to (1) construct high-density and high-quality genetic linkage maps, (2) improve the mapping accuracy of candidate genes for economic traits by combining QTL mapping and RNA-seq, and (3) investigate the stability of QTLs across different environments and explore the impact of environmental effect on the QTL mapping progenies.

## 2 Materials and methods

### 2.1 Plant material and phenotyping

**Figure 1:**
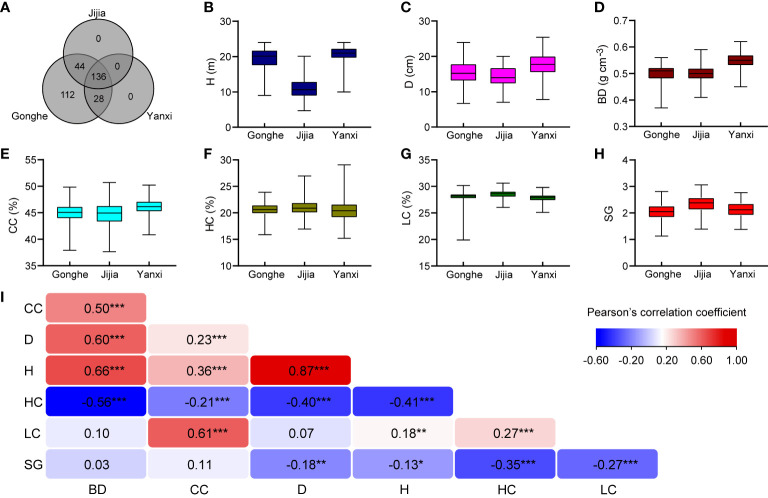
Phenotypic data summary. **(A)** The distribution of clonal progenies among three sites. Boxplots (phenotypic data) of H **(B)**, D **(C)**, BD **(D)**, CC **(E)**, HC **(F)**, LC **(G)**, SG **(H)** of *E*. *urophylla* × *tereticornis* hybrids from Gonghe, Jijia and Yanxi. **(I)** Pearson’ correlation (*r*) coefficients between across-sites genotype best linear unbiased predictions (BLUPs) for pairs of traits and significance of two-sided test (***, ** and * are *P *< 0.001, *P *< 0.01 and *P*<0.05). BLUPs for each genotype, shown as bar plots in **Figure S1**, are given as deviations from the grand mean for each trait (**Table S3**).


The mapping population was developed from a control-pollinated cross between two excellent genotypes, *E. urophylla* (female, genotype UX-30) and *E. tereticornis* (male, genotype T43-05). Both were selected for rapid growth and pulp fibre property traits including lignin, cellulose, and hemi-cellulose. A total of 320 *E. urophylla × tereticornis* F1 hybrid clonal progenies resulting from the cross was described in detail in a previous report (Yang et al., 2018a). Replicated field plantings (at least three ramets per clone per site) including all 320 clonal, full-sib progenies at Gonghe (GH, 112°51’ E, 22°34’ N), and subsets of 180 and 164 progenies at Jijia (JJ, 101°01’ E, 20°21’ N) in Guangdong Province, and Yanxi (YX, 117°52’ E, 24°46’ N) in Fujian Province, China, respectively, were established in April 2006 (**Figure 1A**). Field trials at all three sites were designed as randomized complete blocks with single-tree plots spaced 2 m × 3 m. The number of block-replicates at Gonghe, Jijia, and Yanxi was four, four, and three, respectively. These trials did not contain the parental genotypes. In January 2014, tree height (H, m) and diameter at breast height (D, cm) were measured, and wood properties including basic density (BD, g cm^−3^), cellulose content (CC, %), hemi-cellulose content (HC, %), lignin content (LC, %), and lignin syringyl-to-guaiacyl ratio (SG) were assessed using near-infrared analysis (Yang et al., 2018a). To assess the selection of trees for RNA sampling, which was carried out on the basis of phenotypic values at Gonghe, data for each trait, pooled across the three sites, was analyzed using a linear mixed individual-tree model of the following form (following Baltunis et al., 2007):

Equation 1 $${\text{y}}_{\text{i}}{\text{=X}}_{\text{i}}{\text{s}}_{\text{i}}{\text{+Z}}_{\text{i}}{\text{g}}_{\text{i}}{\text{+Z}}_{\text{i}}{\text{b}}_{\text{i}}{\text{+Z}}_{\text{i}}{\text{r}}_{\text{i}}{\text{+e}}_{\text{i}}$$

where for each trait, ***y_i_*** is a vector of phenotypic measurements on each trait indexed (***i***) by trial for single trait analyses across sites or by trait for bivariate analyses; ***b_i_*** is a vector of fixed effects; ***s_i_*** and ***g_i_*** are vectors of random-effect estimates for genetic and within-site spatial effects, respectively; and ***e_i_*** is a vector of random residual effects. ***X_i_*** is the incidence matrix relating the observations in ***y_i_*** to the fixed effects for mean and site in ***b_i_***:


Equation 2 $${\text{X}}_{\text{i}}{\text{s}}_{\text{i}}=\left[\begin{array}{c} {\text{X}}_{1}\\ 0 \end{array}\begin{array}{c} 0\\ {\text{X}}_{2} \end{array}\right]\left[\begin{array}{c} {\text{s}}_{1}\\ {\text{s}}_{2} \end{array}\right]$$

*g_i_* is the vector of random genetic effects of individual genotypes ***~MVN*** (0, **G⊗A**) where


Equation 3 $$\text{G}=[\begin{array}{cc} {\overset{\wedge }{\sigma }}_{g1}^{2} & \overset{\wedge }{\sigma }g1g2\\ \overset{\wedge }{\sigma }g1g2 & {\overset{\wedge }{\sigma }}_{g2}^{2} \end{array}]$$

and A is the pedigree-based numerator relationship matrix, *Z_i_* is the incidence matrix relating the observations in y_i_ to the random genetic effects in ***g_i_***, ${\hat{\sigma }}_{g1}^{2}$ is the genetic variance and $\overset{\wedge }{\sigma }g1g2$ is the genetic covariance across sites. A comprised elements equivalent to twice the coefficient of kinship (*Ɵ*); with 2*θ* = 0.5 on the off-diagonals and 1+*f* on the diagonal (where *f* is each individual’s inbreeding coefficient, assumed to be zero), reflecting that each clonal progeny is resultant of a controlled pollination between two parents. Vector *b_i_* comprised random effects of complete block replicates within sites $∼MVN\left(0,I_{bi}\sigma_{bi}^{2}\right)$ where ***I_bi_*** is an identity matrix corresponding to the number of blocks and ${\text{σ}}_{\text{bi}}^{2}$ is the variance associated with the block replicates and analogously, ***r_i_*** is a random term associated with the planting rows within sites $∼MVN\left(0,I_{bi}\sigma_{bi}^{2}\right)$ where ***I_ri_*** is an identity matrix corresponding to the number of rows and ${\text{σ}}_{\text{bi}}^{2}\;$ is the variance associated with the rows. Term ***e_i_*** is the random vector of residuals.


Equation 4 $$\text{∼MVN}\left(0\text{, }\left[\begin{array}{c} {\text{I}}_{1}{\text{σ}}_{e1}^{2}\\ 0 \end{array}\begin{array}{c} 0\\ {\text{I}}_{2}{\text{σ}}_{e2}^{2} \end{array}\right]\right)$$

Where ${\text{σ}}_{e1}^{2}\;$ is the residual variance of each trait, ***I_1_*** is the identity matrix corresponding to the number of trees at each site in the case of analysis of a single trait and 0 is the null matrix. For bivariate (trait-trait) correlations, correlated residuals were assumed.

The significance of the genotype effect was gauged by performing additional model runs omitting the term and comparing the Akaike Information Criterion between runs. The mixed models were solved using restricted maximum likelihood implemented in Asreml 4.2 (VSN International, Hemel Hempstead, UK). Bivariate genetic correlation estimates (***r_G_***) between traits ***x*** and ***y*** were obtained from the estimated genetic covariance and variance components as:


Equation 5 $$r_{G}=\frac{{\hat{\sigma }}_{g_{x}g_{y}}}{\sqrt{\sigma_{g_{x}}^{2}\sigma_{g_{y}}^{2}}}$$

where $\overset{\wedge }{\sigma }g_{x}g_{y}$ is the genetic covariance component between traits, and ${\text{σ}}_{{\text{g}}_{\text{x}}}^{2}$ and ${\text{σ}}_{{\text{g}}_{\text{y}}}^{2}$ are the genetic variance components for traits *x* and *y* respectively. These correlations were modelled using Asreml with simultaneous estimation of covariance, and separate genetic and error variances for each trait (see Butler et al., 2018). As solving the bivariate genetic correlation models was problematic for many combinations of traits, Pearson correlations between best linear unbiased predictions (BLUP) for pairs of traits on each genotype were additionally carried out using the FCORRELATION Procedure in Genstat 22 (VSN International, Hemel Hempstead, UK).

### 2.2 GBS library construction, sequencing, and SNP genotyping

Healthy leaves from 320 progenies in Gonghe population and the parental genotypes were collected. An improved CTAB method (Gan et al., 2003) was used for DNA extraction. The GBS library was constructed following the protocol of Poland et al. (2012), with some modifications. This involved screening and optimization of novel enzyme digestion combinations (MspI-MseI). The concentration of GBS library was quantified using a QUBIT fluorometer 3.0 (ThermoFisher, USA), and the size was assessed using the Labchip GXII Touch 24 (Perkin Elmer, USA). The qualified libraries were subjected to paired-end sequencing (PE 150) using the HiSeq 2500 platform (Illumina, USA). Low-quality reads (Q20<20, length<36bp) were subsequently filtered using Trimmomatic v0.36 software (Bolger et al., 2014). Reads were demultiplexed using the “process_radtags” programs of Stacks v2.49 (Rochette et al., 2019). After demultiplexing the raw data, construction of the Stacks catalogue, SNP calling, and genotype construction were performed for reference pipelines using Stacks ref_map.pl program aligned to the *E. grandis* reference genome v2.0 (https://phytozome.jgi.doe.gov/info/Egrandis_v2_0). SNP variants were annotated with SnpEff v4.3 (Cingolani et al., 2012). Finally, segregating SNP markers for a biparental control-pollinated mapping population with phase information from the parental genotypes were exported in the JoinMap v4.1 (Van Ooijen, 2006) format using the “genotypes” program. As a testcross between two heterozygous diploid parents that have resulted from the eucalypt mixed mating system, SNPs were defined as belonging to one of three segregation types: loci that segregate in a 1:1 ratio including those heterozygous in the male parent (*E. tereticornis*) and homozygous in the female (*E. urophylla*) and those heterozygous in the female parent and homozygous in the male); and those heterozygous in both parents segregating in a 1:2:1 ratio (see Maliepaard et al., 1997; Wu et al., 2002 for additional theory).

### 2.3 Genetic linkage map construction

The linkage map was constructed using JoinMap v4.1 and Lep-Map3 (Rastas et al., 2013). Firstly, JoinMap v4.1 was used to divide markers into groups using a grouping tree function in cross-pollinated model with LOD = 12.0 (Van Ooijen, 2006). Subsequently, Lep-Map3 was used to construct the parental genetic maps (Rastas et al., 2013). The process was as follows: Step 1: ParentCall2 was used to call parental genotypes, followed by the Filtering2 step which filtered markers based on high segregation distortion. The default value of the data tolerance (*P*=0.01) was used to discard highly segregated markers (χ^2^ test, *P*<0.01). Step 2: the SeparateChromosomes2 module was used to assigned markers into linkage groups (LGs) by computing all pairwise LOD scores between markers and joined markers with LOD scores range from four to ten. Step 3: OrderMarkers2 module was used to order the markers within each LG by maximizing the likelihood of the data for alternative orders. The Kosambi mapping function was used to estimate map distances. The consensus map for both the maternal and paternal maps was computed using the combine group with the help of shared markers using MergeMap software (Wu et al., 2011).

### 2.4 QTL mapping

QTL mapping based on the consensus map was performed using MapQTL v6.0 (Van Ooijen, 2009). The 95% genome-wide significance LOD threshold was determined by Permutation Test with 10,000 permutations. An initial round of regression Interval Mapping was used to scan putative QTLs with a step size of 1 cM. Linkage groups with peaks near or above the significance threshold were chosen for rounds of automatic cofactor selection for Multiple QTL Mapping with a step size of 0.1 cM. For each round of Multiple QTL Mapping, only cofactors within a significant LOD peak were retained, and Multiple QTL Mapping was performed until the QTL position stabilised to determine the true QTLs. QTL intervals with LOD score greater than the thresholds of LG location during the first round of Permutation Test were declared significant. Simultaneously, the position, genetic effects, and percentage of phenotypic variation explained by the QTL were estimated at the peak of significant LOD score. In order to explore the stability of QTLs across the three sites with contrasting environments, QTLs for seven traits were independently detected for each site. QTLs with overlapping genomic regions, detected at different sites for the same trait, were defined as stable QTLs. All figures were drawn using TBtools v1.046 (Chen et al., 2020).

### 2.5 Transcriptome sequencing and analysis

For each trait (H, D, BD, CC, HC LC, and SG group with H and D sharing a group due to the high correlation between them), six individuals with contrasting phenotypes from 320 progenies of Gonghe population were selected for transcriptome analysis (**Table S1**). These individuals were selected on the basis of phenotypic measurements made at that site. For example, in the BD group, we selected three individuals with low wood density (0.4431, 0.4374 and 0.4508 g cm ^-3^) and three individuals with high wood density (0.5417, 0.5463 and 0.5506 g cm ^-3^), respectively. Due to the overlap of some individuals between trait groups, we selected a total of 23 individuals with contrasting phenotypes, and information on these individuals is shown in **Table S1**. The developing xylem tissues of 23 progenies were collected for RNA extraction in mid-July 2018 which is the period for most-active tree growth in Guangdong, China. In a parallel study, 17 of 23 progenies were used to study the effects of gene expression and alternative splicing on wood formation in *Eucalyptus* (Zhu et al., 2022). The RNA-seq libraries of 23 progenies with contrasting phenotypes were constructed using VAHTS™ Stranded mRNA-seq Library Prep Kit (Vazyme Biotech, China) and were sequenced using the Nextseq-500 platform (Illumina, USA). The raw reads were filtered by removing adaptors and low-quality reads using Trimmomatic v 0.36. Clean reads were mapped to the *E. grandis* reference genome using HISAT2 (Kim et al., 2015), and StringTie v2.0.4 and Ballgown were used to compute the abundance of the transcripts and identify expressed genes and transcripts in each sample (Pertea et al., 2016).

### 2.6 Candidate gene identification and functional analysis

Genes located within the QTL confidence interval were extracted according to the positions of the two closest SNPs in the *E. grandis* reference genome. Then, for each phenotyped trait (H, D, BD, CC, HC, LC, and SG), differentially expressed genes were identified using DESeq2 (Love et al., 2014) with Log2FC ≥ 1 and a false discovery rate (FDR) corrected with *P* ≤ 0.05. For each trait, genes that were differentially expressed in the progenies and those that were mapped on QTLs of the target trait were assumed to be candidate genes. Gene ontology (GO) enrichment analysis was then applied to the biological processes, molecular functions, and cellular components of the candidate genes using PlantRegMap (Tian et al., 2019) with a threshold of *P ≤* 0.05. Kyoto Encyclopedia of Genes and Genomes (KEGG) pathway analysis using Kobas v3.0 (Xie et al., 2011) with a threshold of *P ≤* 0.05. Transcription factors (TFs) were predicted for candidate genes using PlantTFDB v4.0 (Jin et al., 2017) with a threshold value of *P* ≤ 0.05.

## 3 Results

### 3.1 Analysis of phenotypic data

Variance component and fixed effect significance, calculated using F statistics based on the method of Kenward and Roger (1997), for each trait, are given in **Table S2**. There were significant genotype-by-site interactions, except for the HC and LC traits. Only H was significantly different among sites (**Table S2**). All traits were significantly different among genotypes, as gauged by the Akaike Information Criterion (AIC) (**Figures 1B–H**; **Table S3**), where the inclusion of an extra term that results in a lower AIC indicates a significant improvement of the model. **Figure S1** gives example histograms showing genotype BLUP values for two traits and the selections that were made. Estimation of pairwise genetic correlations was problematic for most combinations of traits, with estimates of 0.69 between H and CC and – 0.54 between HC and CC being the only combinations resulting in model convergence and that were estimated with acceptable precision (**Table S4**). This result might be expected, given the small dataset: it is well-established that estimation of trait-trait correlations requires considerable data (e.g. Visscher, 1998). As an alternative, Pearson’ correlations between across-site genotype BLUPs are presented in **Figure 1I**. Pearson’s correlations between across-sites genotype BLUPs revealed strong, positive correlation between H and D, weak negative correlations between SG and H, D, HC and LC and weak to moderate correlations among most other traits with some correlations involving BD being non-significant (**Figure 1I**).

### 3.2 SNP calling and genotyping

**Figure 2:**
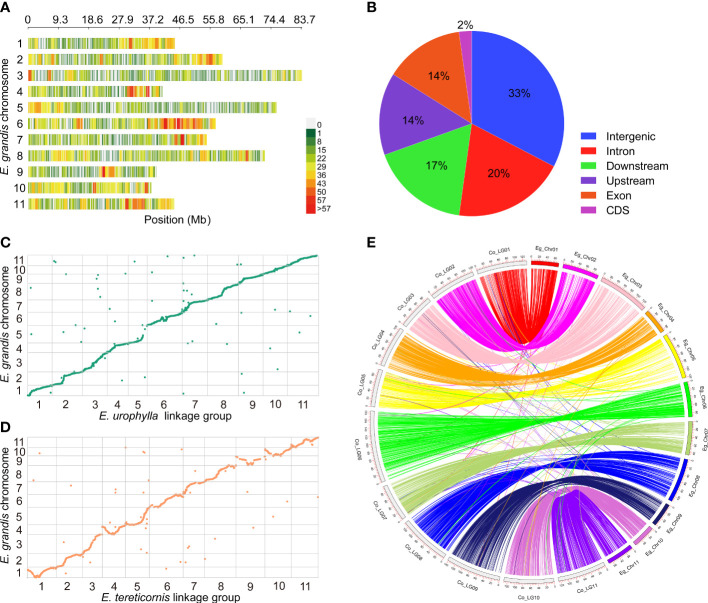
SNP calling and genetic mapping construction. **(A)** Distribution of SNP loci on the chromosomes of *E*. *grandis*. **(B)** Genomic position of SNP loci. Syntenic and colinear relationships of each LGs of *E*. *urophylla* **(C)** and *E*. *tereticornis* **(D)** with *E*. *grandis* genome sequence. In **(E)**, the curve with the same color represents a similarity match between the chromosomes of *E*. *grandis* (e.g., Eg_Chr01) and the corresponding linkage groups of the consensus map (e.g., Co_LG01).

GBS generated about 386 Gb of raw data, comprising 1.6 Gb from the *E. urophylla*, 1.3 Gb from *E. tereticornis*, and an average of 1.2 Gb from the 320 progenies (**Table S5**). The GBS seq data are available at NCBI with accession number PRJNA913962. After processing, using process_radtags in Stacks to demultiplex and discard low-quality reads, 7,336,502 clean reads with mean Q20 of 96.6%, mean Q30 of 89.4% and GC content of 41.7% were obtained from the 320 progenies (**Table S5**). A total of 15,185 high-quality SNPs remained after filtering. Among them, 12,856 (84.7%) SNPs were successfully mapped to the 11 chromosome scaffolds of *E. grandis*, the remainder being located to as-yet unlocated scaffolds on the *E. grandis* genome, with the largest number of SNP variants being found on chromosome 6 (**Figure 2A**). Most SNP variants were observed in intergenic regions (33%), followed by introns (20%), with the fewest in CDS (2%) (**Figure 2B**).

### 3.3 High-density genetic map construction

**Figure 3:**
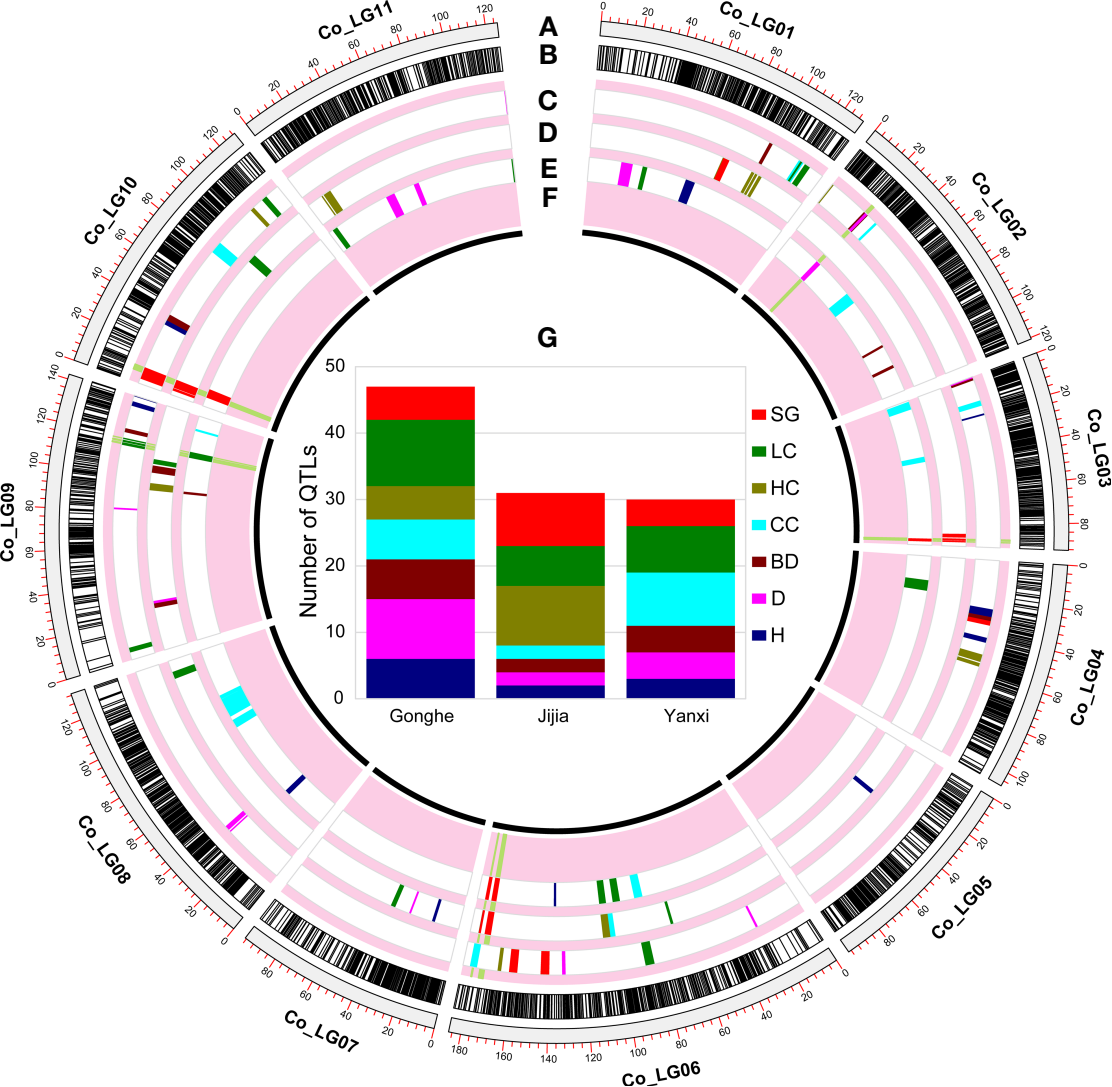
QTL mapping in multiple environments. **(A)** are the genetic distance in cM. **(B)** are the distribution of all loci in the consensus map. And the distribution of QTLs of growth and wood properties detected in Gonghe **(C)**, Jijia **(D)** and Yanxi **(E)** environments. **(F)** are stable QTLs. **(G)** are QTL statistics for each trait at each environment.

Analysis in Lep-Map3 indicated that some markers are effectively redundant, being situated at same locus. This can occur due to close physical location or low recombination rate. Following processing in Lep-Map3, 3686 loci (1615 unique loci) with a total length of 1093.48 cM and a mean interval of 0.68 cM were included in the *E. urophylla* map, while 2463 loci (1167 unique loci) were present in the *E. tereticornis* map, with a total length of 1099.18 cM and an average interval of 0.94 cM (**Table 1**; **Table S6**). Overall, the *E. urophylla* map (1093.48 cM) included 448 more loci than the *E. tereticornis* map (1099.18 cM), but the total genetic map size was very similar (a difference of only 5.70 cM). To further assess the consistency, we BLAST searched the flanking sequences of mapped markers against the *E. grandis* reference genome (**Figures 2C, D**). The number of unique loci that could be mapped to the *E. grandis* reference genome in the *E. urophylla* and *E. tereticornis* maps was 1468 (90.9%) and 1064 (91.2%), respectively, and the number of non-syntenic loci are only 47, and 33, respectively, in these two maps (**Figures 2C, D**; **Table S6**). The results revealed a high degree of synteny between both the *E. urophylla* and *E. tereticornis* genetic maps and the *E. grandis* reference genome. The consensus map merged using MergeMap contains 2754 loci with a total length of 1359.18 cM, and the shortest and longest linkage groups were Co_LG03 (92.39 cM) and Co_LG06 (185.61 cM), respectively (**Table 1** and **Figures 3A–B**). Compared with the parental maps, the marker density and map quality of the consensus map were further improved (**Table 1** and **Figure 2E**).

**Table 1:** Locus statistics of *E. urophylla* map, *E. tereticornis* map and consensus map.

Map	LG	No. of loci	No. of unique loci	Length (cM)	Mean interval (cM)	Max. gap (cM)
*E. urophylla* map	1	283	132	92.08	0.70	3.23
	2	405	178	114.82	0.65	4.92
	3	444	162	93.70	0.58	3.56
	4	277	120	78.80	0.66	4.24
	5	122	78	71.55	0.92	7.76
	6	499	200	132.30	0.66	2.57
	7	269	120	96.76	0.81	3.56
	8	489	182	116.39	0.64	4.58
	9	290	138	92.36	0.67	4.24
	10	278	144	92.81	0.64	2.89
	11	330	161	111.91	0.70	3.56
	Total	3686	1615	1093.48	0.68	–
*E. tereticornis map*	1	242	120	109.40	0.91	5.62
	2	241	120	98.45	0.82	4.58
	3	156	90	74.05	0.82	5.27
	4	208	97	88.51	0.91	3.56
	5	214	102	89.82	0.88	4.24
	6	343	149	128.17	0.86	5.97
	7	215	88	80.56	0.92	9.62
	8	288	129	119.14	0.92	5.62
	9	120	71	109.77	1.55	16.51
	10	241	100	99.32	0.99	8.13
	11	195	101	102.00	1.01	4.58
	Total	2463	1167	1099.18	0.94	–
Consensus map	1	248	248	130.30	0.53	3.56
	2	295	295	120.71	0.41	2.24
	3	250	250	92.39	0.37	2.24
	4	216	216	106.53	0.49	4.24
	5	177	177	97.38	0.55	2.57
	6	344	344	185.61	0.54	5.97
	7	207	207	96.64	0.47	2.89
	8	308	308	132.76	0.43	5.62
	9	208	208	142.22	0.68	8.87
	10	242	242	129.01	0.53	4.01
	11	259	259	125.63	0.49	3.56
	Total	2754	2754	1359.18	0.49	–

### 3.4 QTL analysis

A total of 108 QTLs associated with growth and wood properties were identified in Gonghe, Jijia and Yanxi sites (**Figures 3C–E**; **Table S7**). The LOD scores of these QTLs ranged from 2.32 to 23.27 and explained 2.2 to 23.6% of the phenotypic variation, and 46.3% of the QTLs (50) had positive additive effect (**Figure S2**, **Table S7**). Of these, the minimum physical interval of QTL was 214 bp (*qBD_YX_2_22.74*), the maximum was 4.8 Mb (*qCC_YX_3_2.13*), and the average was 381.97 kb. Detected QTLs were most frequently associated with LC (23), followed by SG (17), CC (16), D (15), HC (14), BD (12) and H (11), with an average of 15.4 QTLs for each trait (**Figure 3G**).

### 3.5 Candidate gene identification and functional analysis

**Figure 4:**
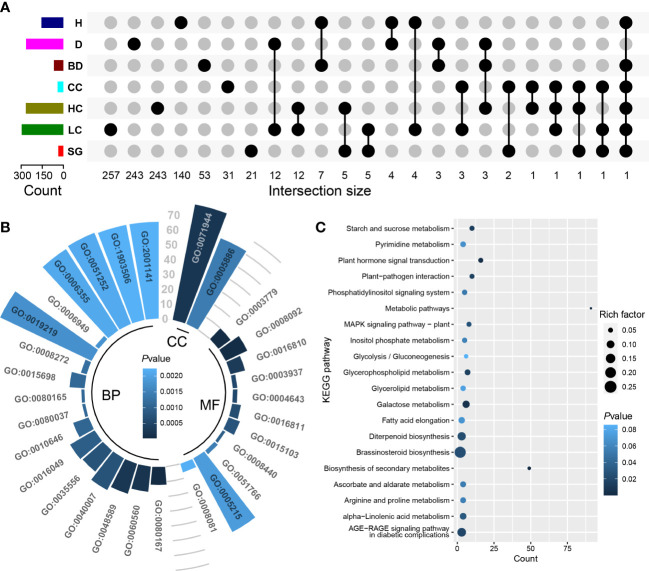
Identification and functional analysis of candidate genes for economic traits. **(A)** Upset plot of the intersection size of candidate genes for each trait. Connected black circles indicate the number of overlapping genes between traits, and unconnected black circles indicate the number of genes unique to a trait. **(B)** Top 30 most-significant GO terms from analysis of candidate genes. The length of the radial bars indicates the gene count. The CC, MF and BP are cellular component, molecular function and biological process, respectively. **(C)** shows the top 20 most-significant KEGG pathways. Details of GO terms and KEGG pathways are shown in **Tables S10**, **S11**, respectively.

**Figure 5:**
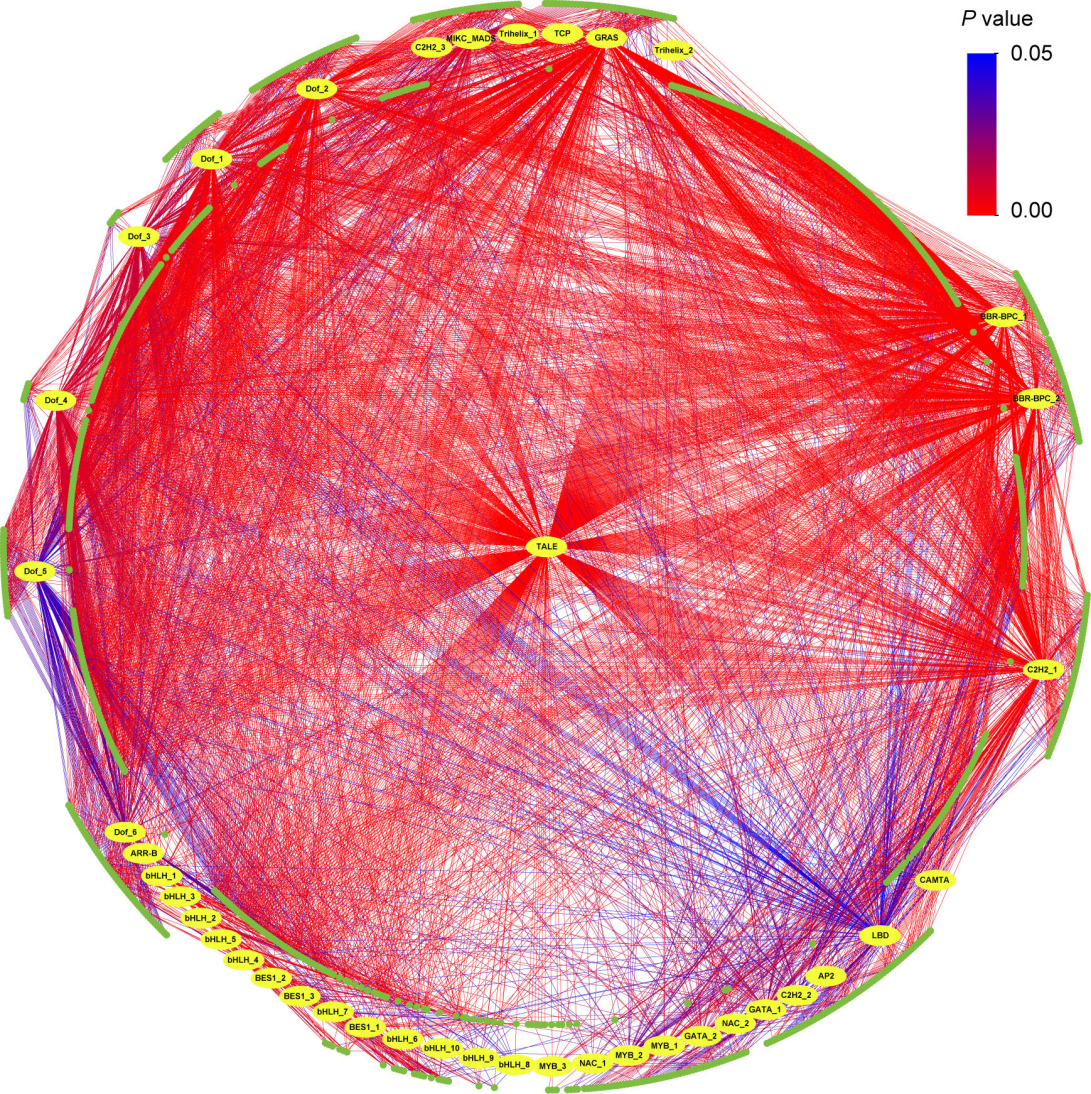
Transcription factor (TF) predicted in candidate genes. Yellow circles are 41 significant TFs and green circles are 944 candidate genes. The color of the line represents the *P* value. Details of TFs are shown in **Table S12**.

Between one (*qBD_YX_2_22.74*) and 798 (*qBD_GH_10_35.72*) genes were identified in the genomic intervals of the aforementioned 108 QTLs (**Table S7**). Based on RNA-seq, totals of 4119 (H and D), 1258 (BD), 813 (CC), 4355 (HC), 2521 (LC), and 937 (SG) differentially expressed genes were identified in each target trait regions, respectively (**Table S8**). One to 73 differentially expressed genes were detected in 95 (88.0% of all QTLs) (**Table S7**). Finally, we identified a total of 1052 candidate genes including some pleiotropic genes that were identified in the QTLs of multiple traits (**Figure 4A**; **Table S9**). GO analysis showed that 831 candidate genes had GO annotation and 229 GO terms were significantly enriched (**Table S10**). The most significant GO terms were response to karrikin (GO:0080167), actin binding (GO:0003779) and cytoskeletal protein binding (GO:0008092) (**Figure 4B**; **Table S10**). KEGG analysis revealed 12 significantly enriched pathways, including plant hormone signal transduction, galactose metabolism, and plant-pathogen interaction, suggesting that these pathways may exert important regulatory effects on economic traits in *Eucalyptus* (**Figure 4C**; **Table S11**). Transcription factor prediction indicated that 944 candidate genes significantly enriched 41 TFs from 17 families (**Figure 5**; **Table S12**). Among them, a large number of candidate genes were encoding TFs found in the Barley B Recombinant-Basic Pentacysteine (BBR-BPC) and Three-Amino-Acid-Loop-Extension (TALE) family.

### 3.6 QTLs that were stable across sites

Ten QTLs were identified that were stable across at least two sites (**Figure 3F**; **Table S7**). Among them, only one QTL, *qSG10Stable*, was stable at all three sites, which was mapped on chromosome 10 for SG, and explained 23.6%, 18.4% and 19.4% of phenotypic variation in Gonghe, Jijia and Yanxi, respectively (**Table S7**). Nine QTLs that were stable across two sites were associated with BD (*qBD2Stable*), D (*qD2Stable*), SG (*qSG3_1Stable, qSG3_2Stable, qSG6_1Stable, qSG6_2Stable*) and LC (*qLC9_1Stable, qLC9_2Stable, qLC9_3Stable*). We also noted that several different QTLs, situated in proximate genomic regions, were detected across different sites. The following examples give the names of QTLs which are a concatenation of the trait code, the site code, the chromosome number and the QTL peak (cM): *qCC_YX_3_2.13* and *qCC_GH_3_15.36*; *qLC_JJ_6_67.99*, *qLC_GH_6_87.31* and *qLC_YX_6_98.12*. Despite the lack of overlap in the QTL intervals detected across sites, they were taken as potentially stable QTLs. A total of 1173 genes, including 31 candidate genes, were annotated in the physical genomic intervals of the nine stable QTLs (**Table S9**).

## 4 Discussion

### 4.1 High density genetic maps of *Eucalyptus* were constructed based on GBS technology

In this study, we constructed genetic maps of *E. urophylla* and *E. tereticornis* and their consensus maps using large-scale SNP markers generated by GBS. The mean marker spacing of our *E. urophylla* map, *E. tereticornis* map and consensus map were 0.66 cM (a total of 1615 SNPs with 1093.48 cM), 0.94 cM (1167 SNPs with 1099.18 cM) and 0.49 cM (2754 SNPs with 1359.18 cM), respectively, representing a considerable increase in marker density and total number of markers mapped, relative to previous maps, constructed using the same populations, employing genomic SSR, EST derived SSR, EST derived CAPS, and DArT markers (Li et al., 2015). These results demonstrate the superiority of current GBS technology in the genetic map construction for *Eucalyptus*. The marker density of our *E. urophylla* map and consensus map were slightly lower than that recently reported in *E. urophylla* (1773 SNPs with 885.9 cM) (Bartholomé et al., 2015) and the consensus map of *E. grandis* × *E. urophylla* (2290 markers with 1107.6 cM) (Kullan et al., 2012), however, the *E. tereticornis* map constructed here is the densest to date (such as, 585 markers with 1241.4 cM in Li et al., 2015; 204 markers with 1023.56 cM in Sumathi et al., 2018). Generally, the density of maps constructed here also compare favourably with recent GBS- or restriction site-associated sequencing (RAD-seq) maps for other forest species. For example, the density of our maps was close to that for *Pinus* (Hirao et al., 2022) and significantly superior to the *Populus* maps (Carletti et al., 2016; Xia et al., 2018; Tong et al., 2020).

Some of the differences in resolution of the genetic maps may be explained by the size of the mapping population (Yang et al., 2018b). For example, 1025 F1 progenies were employed in the previous *E. urophylla* map (Bartholomé et al., 2015), compared with 320 F1 progenies in ours and 130 F1 offspring in *Pinus* map (Hirao et al., 2022), and 131 to 300 F1 progenies in *Populus* maps (Carletti et al., 2016; Xia et al., 2018; Tong et al., 2020). Although preserving large mapping populations is difficult and rarely implemented in forest trees, it may contribute to the accuracy and reliability of genetic linkage mapping and subsequent QTL mapping.

It is also noted that larger and more-complex genomes make SNP calling and genetic mapping more difficult. For example, the genome of *Pinus* is very large – *P. taeda* is around 20 Gb - and complex, including repetitive sequences, transposable elements, and gene duplication (Zimin et al., 2014). In contrast, we used a simplified strategy for SNP calling in *Eucalyptus*, which possesses a much smaller genome, using a combination of double digests. The higher resolution of the previous *E. urophylla* map may have benefited from the greater number of genome-wide SNP variants detected by whole-genome resequencing technology (Bartholomé et al., 2015). Similarly, ultra-high-resolution genetic linkage maps were reported in several crops based on whole-genome resequencing (Luo et al., 2020; He et al., 2021; Kong et al., 2022). It should be noted that the map resolution is only one of the possible indicators of map quality, considering the differences in genome size and complexity among different genera, the differences in algorithms and functions of different mapping software, and the effects of biased separation markers.

While GBS technology provides very numerous markers, enabling construction of relatively high-density genetic maps, due to the markers being unevenly distributed within the genome (**Figure 2**), regions of low marker density still exist. For example, we detected a gap of 16.51 cM in Et_LG09 91.37–107.88 cM and a gap of 8.87 cM in Co_LG09 5.51–14.38 cM (**Tables 1** and **S6**). These low density regions may be caused by low polymorphism of genetic variation (Kullan et al., 2012). Alternatively, the efficiency of restriction enzyme digestion selected in GBS varies in species (Wang et al., 2017), which may result in low genome coverage. Higher depth, simplified genome sequencing complemented by whole genome resequencing with broader coverage may be a strategy that provides better uniformity and higher density. In addition, the selection of appropriate restriction enzymes can also improve the SNP calling efficiency. It is worth remarking that the type II restriction enzymes (e.g., MspI-MseI) used in this study tend to result in low coverage of the methylation regions of the genome (Hashimoto et al., 2007). Whether or not the low marker density regions in our genetic map correspond to abundant methylation regions deserves further investigation. In future, high-depth coverage and analysis of all genomic regions might be done through development of methylation markers that can be combined with the aforementioned strategies of high depth, simplified GBS and whole-genome resequencing. Overall, our results confirm that the low-cost of GBS technology provides tens of thousands of genomic loci, significantly improving the genetic map resolution of *Eucalyptus*.

### 4.2 QTL mapping and candidate gene identification of growth and wood properties

A total of 108 QTLs associated with economic traits were identified at three sites, and the number of QTLs for most traits was higher than those identified in previous studies (Freeman et al., 2009; Gion et al., 2011; Li et al., 2015; Sumathi et al., 2018). Taking wood density as an example, Freeman et al. (2009) detected three QTLs on three LGs, Li et al. (2015) detected four QTLs on four LGs, and we detected 12 QTLs on six LGs. Most of the 12 QTLs were not identified in previous studies, suggesting that high-density genetic mapping reveals more QTLs including some that are novel. Moreover, in further comparison, the phenotypic variance explained (PVE) of the previous wood density QTLs ranged from 9.5 to 15.5 (Freeman et al., 2009; Li et al., 2015), while the average PVE of our 12 QTLs was only 4.9, with a maximum of 11.2 (*qBD_YX_2_106.18*). This indicates that that the economic traits of *E. urophylla* × *tereticornis* may be regulated by numerous QTLs, each of small effect, which accords with the assumption that these are quantitative traits.

QTL mapping and RNA-seq are two different approaches that focus on the identification of trait-related genes at different regulatory stages. Target trait genes identified by QTL mapping tend to be more involved in upstream molecular regulation (He et al., 2021). In contrast, due to the transient nature of RNA-seq, more of the genes that are involved in regulation tend to be identified (Wang et al., 2018). In addition, some studies have used post-metabolic products to infer the genes involved in regulation (Galpaz et al., 2018; Peng et al., 2022). Our results showed that 88.0% of the QTLs were involved in transcriptional regulation (**Table S7**), indicating that the results of QTL mapping and RNA-seq can be efficiently inter-validated.

Examination of known gene function indicates that many of the 1052 candidate genes have plausible links to the regulation of economic traits in *E. urophylla* × *tereticornis* (**Table S9**). These candidate genes are mainly associated with sugar metabolism, signal transduction, morphogenesis and response, as well as several known wood biosynthetic pathways (Zhang et al., 2016). We found that many candidate genes are involved in the encoding of TFs (**Figure 5**). For example, TALE, a class of homologous structural domain proteins, has been shown to play an important role in the secondary growth of plant cell walls and the development of root and stem (Magnani and Hake, 2008). In this study, 479 candidate genes encoding TALEs were identified.

In addition to genes encoding TALEs, some TFs were also found. MYB is a class of plant-specific TFs in the plant genome. In *Arabidopsis thaliana*, MYB46 and MYB83 have been confirmed as secondary master switches regulating secondary wall formation, and overexpression of both genes results in ectopic secondary wall deposition (Zhong et al., 2007; McCarthy et al., 2009). In *Populus*, more than 15 MYBs have been reported to be involved in secondary wall formation, of which six were confirmed to be secondary master switches (Li et al., 2020a). In the present study, 105 candidate genes encoding three MYBs were also detected (**Table S12**). We hypothesize that these candidate genes may have a switch function similar to that in *Populus*, regulating the wood formation process in *Eucalyptus*. In addition, TFs of the NAC, Lateral Organ Boundaries Domain (LBD), DNA binding with one finger (Dof) and Basic Helix-Loop-Helix (bHLH) and other families have been reported to be involved in the growth and wood formation process (Yordanov et al., 2010; Hussey et al., 2011; Xiang et al., 2019; Sun et al., 2021). Our results suggest that these candidate genes may have important effects on economic traits by encoding TFs, and those genomic resources for further research will accelerate breeding for economic traits in *Eucalyptus* and also help to reveal the genetic basis of wood formation.

### 4.3 Stable QTLs

In the context of a considerable number of *Eucalyptus* clones that have been successfully used in production forestry, screening for stable QTLs among specific hybrid progeny is a realistic strategy to guide practical breeding. Therefore, we used clonally reproduced full-sibs of a hybrid deployed across sites to carry out QTL stability studies. This is an important difference between the present and previous studies (Freeman et al., 2011; Freeman et al., 2013). The use of clonal replication within and across sites reduces the effect of genetic variation on phenotypic variation and thus allows better partitioning of environmental variance. Among 108 QTLs, the vast majority were not stable across the three study sites, which is consistent with previous findings in *E. globulus* (Freeman et al., 2013), *C. japonica* (Mori et al., 2019; Mori et al., 2020), *P. taeda* (Westbrook et al., 2015), and some crops (Raihan et al., 2016; He et al., 2021). These studies suggested that quantitative traits are influenced by interactions between genetic and environmental factors, resulting in the identification of specific QTLs in specific environments only.

Of the ten QTLs detected at more than two sites (**Figure 3F** and **Table S7**), only *qSG10Stable* was stable across all three sites, mapped between positions 29,512,351 and 30,943,933 bp of chromosome 10. It is interesting to note that the two significant SG loci identified on chromosome 10 (30,915,259 bp) in *E. globulus* (Cappa et al., 2013) were located exactly in the *qSG10Stable* interval. Similarly, Mizrachi et al. (2017) identified two eQTLs associated with LC on chromosome 10, from which a large number of genes associated with the lignin biosynthetic process were identified. In *qSG10Stable*, we identified two candidate genes (*Eucgr.J02456*, *Eucgr.J02459*) encoding glycosyl hydrolase family 38 proteins (**Table S9**). Previously, Wei et al. (2017) integrated RNA-seq and GWAS analysis and identified one candidate gene encoding a glycosyl hydrolase that regulates the LC of *Brassica napus.* This evidence suggests a strong association signal between *qSG10Stable* and lignin biosynthesis.

We found that *qSG10Stable* explained the most phenotypic variation at all three sites (PVE: 23.6% in Gonghe, 18.4% in Jijia, 19.4% in Yanxi), suggesting that QTLs with strong effects have higher likelihood of stability across environments, a finding supported by previous studies (Wan et al., 2005; Raihan et al., 2016; He et al., 2021). However, the PVE of stable QTLs may change in different environments due to the interaction between QTL and environment, for example, the PVEs of the other nine partially stable QTLs (i.e. those stable across two sites only) are highly variable. We found only one partially stable QTL, *qD2Stable*, associated with growth traits. This might be expected, as growth traits tend to be subject to stronger QTL-by environment interactions than wood properties (Bartholomé et al., 2013; Freeman et al., 2013; Mori et al., 2019).

Climatic factors may have important effects on the QTL stability across environments. For instance, in a multi-environment (two relatively drier and one wetter regions) QTL study of *C. japonica*, two stable QTLs were detected only in the two drier regions and not in the wetter region, and their stability was considered to be related to limited water availability during the growing season (Mori et al., 2019). Our study sites included two subtropical monsoon climates, Gonghe and Yanxi, and one tropical maritime climate, Jijia, which are climatically significantly different (Yang et al., 2018a). It would therefore seem likely that studies involving more sites or sites with widely varying climates would identify even fewer QTLs that are stable across all sites. However, such studies, although expensive to establish, may allow us to better assess the nature of QTL × environment interactions and potentially identify QTLs that are at least partially stable across a predictable subset of site types. Moreover, as most of these stable QTLs are associated with pulp traits, they are valuable for future breeding of *E. urophylla* × *tereticornis* hybrids.

## Data availability statement

The GBS seq data has been deposited at NCBI with accession number PRJNA913962.

## Author contributions

XZ: performed molecular lab work and wrote the manuscript with assistance from all other authors; QW, CZ, DB: phenotype traits measurement and analysis; QW, CZ, FL: maintained the field trial and collected the leaf samples; XZ, DB, HZ, PW, FL: analyzed the data; DB: review and editing the manuscript; FL: conceived and designed the project, review and editing the manuscript. All authors read and approved final manuscript.

## Supplementary material

Supplementary Figure 1 Histograms showing best linear unbiased prediction deviations (across-sites genotype values) for inter-specific hybrid eucalypt full-sib progeny from across-sites mixed model analyses for HC and SG traits. Red bars indicate individuals selected on the basis of their above- or below-average genetic values. Some genotypes included more than one selected individual.

Supplementary Figure 2 LOD scores for linkage groups of consensus map for each trait in three environments. The LOD thresholds of QTL are given in **Table S7**.

Supplementary Table 1 Twenty-three individuals with contrasting phenotypes for RNA-seq.

Supplementary Table 2 Analysis of linear mixed models. Random effects by trait for block-replicate and row (both within site), genotype, genotype-by-site interaction (GxS) and heterogeneous site residuals and fixed-effect F statistic, denominator degrees of freedom (d.d.f.) and F probability for the fixed site term. For each variance component estimate, Z (the component divided by it standard error) is given. Informally, Z>2 indicates a term that is likely to be gauged significant using the AIC. **Con.* Variance component was constrained to zero with zero standard error.

Supplementary Table 3 Akaike Information Criterion and grand means from mixed model analyses (means net of block, column and genotype BLUP effects) for each trait.

Supplementary Table 4 Bivariate genetic correlations between pairs of traits with correlation estimates (lower triangle - B denotes a boundary estimate) and Z (upper triangle - the estimated correlation coefficient divided by its standard error– given where estimable).

Supplementary Table 5 Data statistics of GBS sequencing.

Supplementary Table 6 Marker information in the genetic maps.

Supplementary Table 7 Information of 108 QTLs and ten stable QTLs for economic traits.

Supplementary Table 8 Differentially expressed genes identified in each trait group by RNA-seq.

Supplementary Table 9 Information of 1052 candidate genes and genes in stable QTLs

Supplementary Table 10 GO enrichment of candidate genes.

Supplementary Table 11 KEGG enrichment of candidate genes.

Supplementary Table 12 Transcription factor prediction of candidate genes.
