# Supplementary figures and images for: High-density genetic linkage mapping reveals low stability of QTLs across environments for economic traits in Eucalyptus
Source: Front Plant Sci. 2023 Jan 18;13:1099705. doi: 10.3389/fpls.2022.1099705 (PMC10112524; doi:10.3389/fpls.2022.1099705)

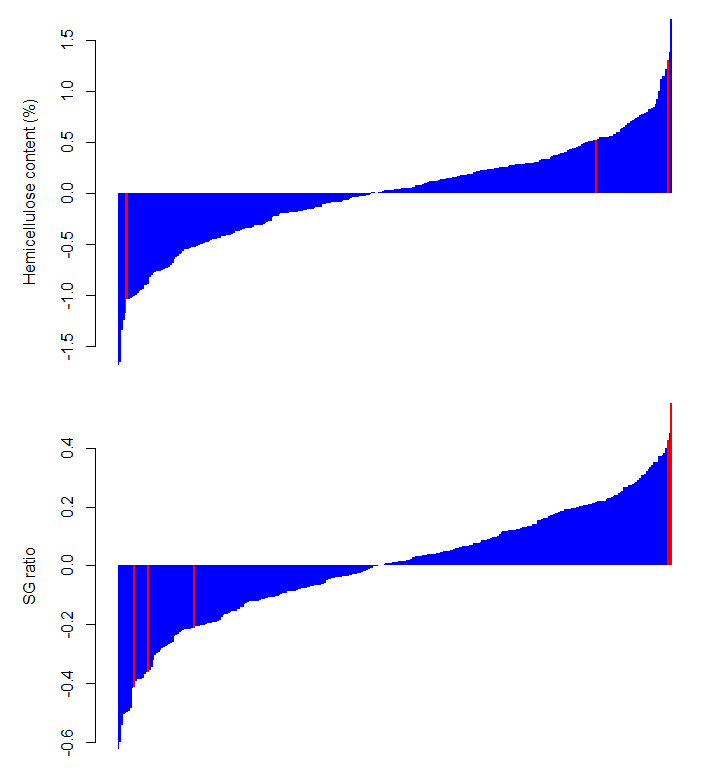

Supplement: Supplementary Figure 1 — Histograms showing best linear unbiased prediction deviations (across-sites genotype values) for inter-specific hybrid eucalypt full-sib progeny from across-sites mixed model analyses for HC and SG traits. Red bars indicate individuals selected on the basis of their above- or below-average genetic values. Some genotypes included more than one selected individual. [file Image_1.png]

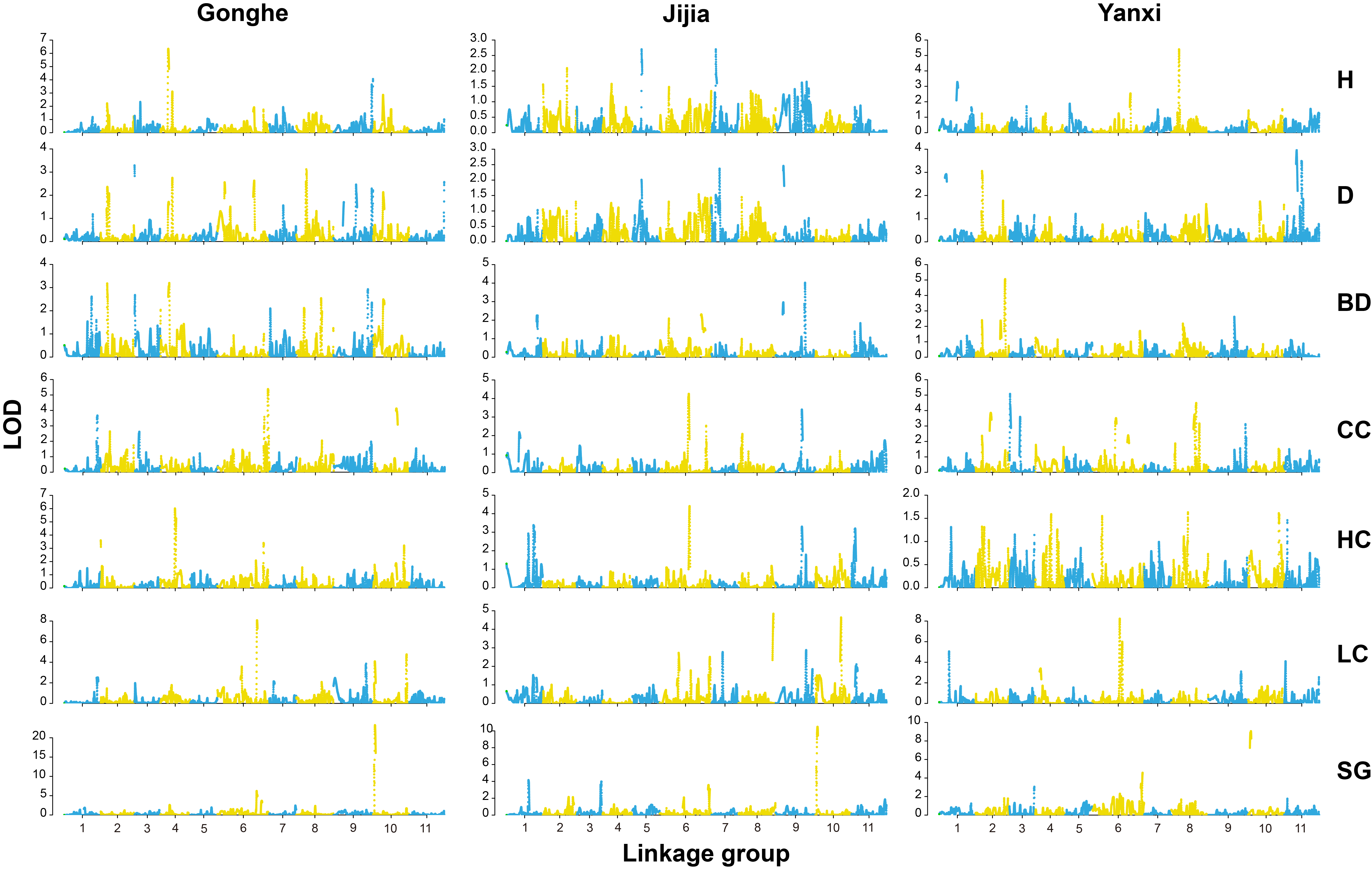

Supplement: Supplementary Figure 2 — LOD scores for linkage groups of consensus map for each trait in three environments. The LOD thresholds of QTL are given in Table S7 . [file Image_2.tif]
